# Comparative Analysis of Transcriptome Profiles in Patients with Thromboangiitis Obliterans

**DOI:** 10.3390/genes15010019

**Published:** 2023-12-21

**Authors:** Gözde Öztan, Nilgün Bozbuğa, Halim İşsever, Fatma Oğuz, İrem Canıaz, Nilgün Yazıksız, Melike Ertan, İbrahim Ufuk Alpagut

**Affiliations:** 1 Department of Medical Biology, Istanbul Faculty of Medicine, Istanbul University, Topkapi, 34093 Istanbul, Turkey; oguzsf@istanbul.edu.tr; 2 Department of Cardiovascular Surgery, Istanbul Faculty of Medicine, Istanbul University, Topkapi, 34093 Istanbul, Turkey; nilgun.bozbuga@istanbul.edu.tr (N.B.); icaniaz@istanbul.edu.tr (İ.C.); nilgun.yaziksiz@istanbul.edu.tr (N.Y.); melike.ertan@istanbul.edu.tr (M.E.); ibrahim.alpagut@istanbul.edu.tr (İ.U.A.); 3 Department of Public Health, Istanbul Faculty of Medicine, Istanbul University, Topkapi, 34093 Istanbul, Turkey; hissever@istanbul.edu.tr

**Keywords:** TAO, Buerger’s disease, microarray analysis, genes, Clariom D array, diagnostic markers

## Abstract

Background: Thromboangiitis obliterans (TAO) causes vascular insufficiency due to chronic inflammation and abrupt thrombosis of the medium and small arteries of the extremities. In our study, we aimed to determine biomarkers for the diagnosis of TAO by evaluating 15 male TAO patients with Shinoya diagnostic criteria and 5 healthy controls who did not have TAO-related symptoms in their family histories. Methods: The Clariom D Affymetrix platform was used to conduct microarray analysis on total RNA extracted from whole blood. A total of 477 genes (FC ≤ 5 or >5) common to the fifteen patient and five control samples were selected using comparative microarray analysis; among them, 79 genes were upregulated and 398 genes were downregulated. Results: According to FC ≤ 10 or >10, in the same TAO patient and control group, 13 genes out of 28 were upregulated, whereas 15 genes were downregulated. The 11 key genes identified according to their mean log2FC values were *PLP2, RPL27A, CCL4, FMNL1, EGR1, EIF4A1, RPL9, LAMP2, RNF149, EIF4G2,* and *DGKZ*. The genes were ranked according to their relative expression as follows: *FMNL1* > *RNF149* > *RPL27A* > *EIF4G2* > *EIF4A1* > *LAMP2* > *EGR1* > *PLP2* > *DGKZ* > *RPL9* > *CCL4.* Using protein–protein interaction network analysis, *RPL9, RPL27A,* and *RPL32* were found to be closely related to EIF4G2 and EIF4A1. The Reactome pathway found pathways linked to 28 genes. These pathways included the immune system, cellular responses to stress, cytokine signaling in the immune system, and signaling by ROBO receptors. Conclusions: By figuring out the protein expression levels of the genes that have been found to explain how TAO disease works at the molecular level, it will be possible to figure out how well these chosen transcripts can diagnose and predict the disease.

## 1. Introduction

Young male smokers from low-income and middle-income countries are more likely to get thromboangiitis obliterans (TAO), which is also called Buerger’s disease. Blockage of intermediate-sized arteries may cause tissue or limb loss [1,2]. TAO is uncommon globally, with an unknown cause and epidemiology [3]. There is no standardized treatment method other than avoidance of tobacco exposure [4]. Angiogenesis is one of the approved therapies for TAO in individuals with and without critical limb ischemia [5]. Although the disease is seen all over the world, it accounts for as little as 0.5–5.6% of all peripheral vascular diseases in Western Europe, whereas this rate is 45–63% in India and as high as 16–66% in Korea and Japan. In Ashkenazi Jews, the rate reaches 80% [6].

The specific cause of the apparent rise in the disease’s incidence among women is unknown; however, women’s higher cigarette usage may explain it [7]. Men and women tend to have similar disease features and prognoses [8]. Although it affects both genders and all races, its incidence is much higher in the male gender. There is no epidemiological study about how often Buerger’s disease happens in the Turkish population. Vascular dysfunction after the endothelial damage initiated by cigarette smoking is thought to play a role in the etiology of thromboangiitis obliterans, along with infectious factors such as vasodilation defects, hypercellular thrombus formation, and rickettsia. Evidence from research suggests that TAO is caused by an inflammatory process that arises due to vulnerability to infection as a consequence of a lowered immune response and aberrant gene expression in the innate immunity system [6,9]. The multifactorial etiology of TAO consists of components such as hereditary tendency, tobacco use, and the immune and procoagulant responses [10]. In recent years, inflammatory responses have been linked to a worsening of Buerger disease’s symptoms. Evidence of this comes from the rise in inflammatory cytokines. It is possible that the inflammatory cytokines contribute to the progression of Buerger disease by interfering with endothelial function. Endothelial dysfunction may be detectable and quantifiable well before the onset of the overt vascular disease and its symptoms [11].

Smoking-induced vascular endothelium damage and an inflammatory reaction cause TAO. Endothelial cells are a key part of how vasculitis lesions form and stay in place, and endothelial dysfunction is shown by a decrease in endothelium-dependent vasorelaxation. Two of the most important molecules involved in mediating intercellular adhesion are intercellular adhesion molecule 1 (ICAM-1) and vascular cell adhesion molecule 1 (VCAM-1) [12]. Diagnosis of the disease involves assessing symptoms, examination findings, imaging methods, and meeting some commonly used diagnostic criteria. None of these criteria alone is sufficient to make a diagnosis. The Shinoya diagnostic criteria and the Olin diagnostic criteria are now the most commonly utilized sets of diagnostic criteria [13,14]. Shionoya’s criteria were used to make a diagnosis of TAO, and digital subtraction angiography (DSA) validated the diagnosis [15]. Focal high-grade stenosis or abrupt vascular blockage with well-established collaterals are the DSA results [16]. In 1996, Papa and Adar developed a scoring system for Buerger’s disease [17]. The clinical criteria proposed by Shionoya were utilized in order to arrive at a diagnosis. These criteria included: (1) a smoking history, (2) an onset that occurred prior to the age of 50 years, (3) infrapopliteal arterial occlusion, (4) either upper limb involvement or phlebitis migrans, and (5) the absence of atherosclerotic risk factors other than smoking [18,19,20].

Diagnosing TAO does not yet have a reliable set of laboratory tests. To rule out other possible causes of symptoms similar to TAO, a full serological panel, including a complete blood count, liver function, kidney function, blood glucose, erythrocyte sedimentation rate, C-reactive protein, antinuclear antibodies, rheumatoid factor, anticentromere antibodies, antiphospholipid antibodies, lipid profile, anti-Scl-70 antibodies, segmental arterial doppler pressures, urinalysis, arteriography, computed tomography, echocardiography, biopsy, complete thrombophilia screening, and radiographs of the hand, should be obtained [21]. In practice, when there is no evidence of major artery atherosclerosis, angiography and vascular laboratory data may be used to diagnose TAO. Patients with antiphospholipid syndrome who show signs of thrombus but no inflammation cannot be diagnosed with TAO from a medical standpoint. Although doppler ultrasonography and angiography may aid in disease diagnosis, the results of these tests do not always indicate the presence of TAO. Although histology is the most reliable method for making a conclusive diagnosis, it is not routinely used in clinical practice because of the risks involved with biopsy and financial concerns [22].

In our study, we aimed to determine biomarkers for the diagnosis of TAO disease by evaluating male patients with a clinically high probability of TAO using the most widely used and accepted diagnostic criteria in the literature. In this study, it is planned to obtain transcriptomic data on TAO disease and identify new candidate biomarkers by analyzing the transcriptome data for patients with thromboangiitis obliterans using bioinformatics methods and tools involving the Clariom D Assay, which are new-generation transcriptome-level expression profiling tools. With the Affymetrix GeneChip Gene Array, which allows the detection of transcript variants and alternative splicing events, molecular information for the determination of the mechanisms of TAO will be defined and the diagnostic and prognostic potentials of selected transcripts will be revealed. Considering that early diagnosis and treatment are very important in this disease, which does not have a standardized treatment protocol, the transcripts identified will have the potential to be diagnostic and prognostic biomarkers for patients with TAO.

## 2. Materials and Methods

### 2.1. Clinical Specimens

In the study, according to the Shionoya criteria, a smoker who applied to the Istanbul Faculty of Medicine Cardiovascular Surgery Polyclinic was randomly selected from among 15 male patients diagnosed with TAO and 5 healthy controls who were randomly selected from blood donors in blood transfusion institutions with no family history of symptoms related to TAO. Among the patients who applied to the outpatient clinic, those who met all the Shionoya diagnostic criteria (smoking history, ischemia findings before the age of 50, infrapoplital occlusion, involvement of the upper extremity arteries, and absence of atherosclerotic risk factors other than smoking) and those who had 6 points or more according to the Papa and Ader diagnostic criteria (age of onset, claudication, upper extremity involvement, migratory superficial vein thrombus, Raynaud color change, angiography, biopsy, age of onset, gender, smoking history, localization, nonpalpable pulses, atherosclerosis (ASO), diabetes mellitus (DM), hypertension (HT), hyperlipidemia (HL)) were accepted as TAO (Table 1). The patients who participated in the study as a control group were evaluated with routine blood tests, and no pathology was detected in the tests. In peripheral pulse examinations, arterial flows are coded as triphasic using a hand Doppler. Patients who do not have symptoms such as claudication, pain, or thrombophlebitis and do not have a history of additional diseases. None of them smoke. In addition, the age distribution of the five male control groups is 43.60 ± 3.51; the median (min–max) is 44 (38–47) and the average age values between the patient and control groups were found to not be statistically significant (independent samples *t*-test).

The major reason statins reduce cardiovascular diseases and atherosclerotic strokes is because they decrease cholesterol levels. This effect has been well established. In addition to their cholesterol-lowering effects, statins may impact cerebrovascular disorders in ways that are not immediately apparent. Statins may slow the onset of cardiovascular disease by reducing inflammation, enhancing vascular endothelial function, stabilizing plaques, and preventing platelet aggregation, among other potential benefits [23]. The effect of the statin dose on pulmonary arterial hypertension (PAH) development, amputations, and quality of life needs more investigation. According to new research, patients suffering from chronic limb-threatening ischemia may find an improvement in lower extremity functioning after receiving intramuscular injections of nanoparticles containing pitavastatin [24].

However, the basic public health approach is to ensure that all healthcare personnel in contact with PAH patients are aware of the high cardiovascular risk associated with this disease and the need to control all risk factors for the patients and reduce negative effects in the future with the secondary protection of this population.

In the patient evaluation forms we prepared, information was obtained about age, gender, smoking history (amount, duration, and age of onset), educational status, demographic characteristics, additional diseases (DM, HT, HL, rheumatological diseases), drug use, and family history. Pain character (clodican, rest, ulcer, necrosis), extremity skin findings (pallor, redness, sensory disturbance, motor loss, atrophy, amputation), distal pulse examination, and ABI (ankle brachial index, arm–ankle index) were measured. From the venous blood samples taken test for fasting blood glucose, lipid profile, antinuclear antibodies, Scl-70, proteins C and S, erythrocyte sedimentation rate, antithrombin III levels, anticentromere antibodies, rheumatoid factor, antiphospholipid antibody factor V Leiden were requested for differential diagnosis. Ethics committee approval was obtained for our study (Istanbul Faculty of Medicine Clinical Research Ethic Committee approval number/date: E-29624016-050.99-252243/22 June 2021). All participants provided their informed permission before taking part.

### 2.2. RNA Isolation

The manufacturer’s protocol was followed to extract the total RNA from whole blood samples using the QiaAmp RNA Blood Mini Kit (catalog no. 52304; Qiagen, Hilden, DE, Germany). The NanoDrop 2000c (Thermo Fisher Scientific, Wilmington, DE, USA) was used to measure the RNA quality and quantity (A260/A280:1.8–2.0).

### 2.3. Affymetrix Microarray Hybridization Analysis

Following the manufacturer’s instructions, high-quality RNA was extracted from 20 separate samples, tagged, and hybridized on human Clariom D gene chips (catalog no. 902922; Affymetrix, Santa Clara, CA, USA). The protocol was as follows: whole RNA was used to make cDNA and cRNA, which were then hybridized for 16 h at 45 °C in an Affymetrix GeneChip 645 hybridization oven, and the results were read off a human transcriptome array 2.0. A GeneChip Fluidics Station 450 was used to dye the arrays. After that, a GeneChip™ scanner 3000 was used to read the data from the chip. DAT files containing the array’s fluorescence signals were collected. Raw data in ARR and DAT image files provided the information about the pixel intensity levels. The Affymetrix GeneChip Command Console software version 4.0.1.36 was used in the process of transforming the raw data contained within the ARR and DAT image files into the intensity data that were included inside the CEL and CHP files. Affymetrix Clariom D.CEL data were normalized using Expression Console software version 1.4.1 to provide probe-level signal expression values, which were then stored as CHP files. The Transcriptome Analysis Console (version 4.0.2) was used to examine the CHP files for the expression of genes, exons, splice variants, and associated pathways involved in TAO formation [25].

### 2.4. Gene Ontology (GO) Enrichment Analysis

GO is a free online resource describing the function of genes in biological systems and pathways and keeping these annotations in a computable format. According to the GO database, genes have biological descriptors (GO terms) derived from the characteristics of the proteins encoded within them. Three types of terms can be categorized: biological process, molecular function, and cellular component. An application based on the R/Bioconductor package called ShinyGO utilizes a large pathway database. GO enrichment results and gene properties are graphically visualized with ShinyGO 0.77, which provides application program interface access to KEGG and STRING.

## 3. Results

### 3.1. Features of Differentially Expressed Genes (DEGs)

DEGs are genes that fulfill both the *p*-value threshold of 0.05 (one-way between-subjects ANOVA, Ebayes) and the fold-change threshold of >10. These genes were included in the gene ontology (GO) study. When comparing TAO patients and healthy controls, we found that the expression of 4970 genes was significantly different. The 4970 genes were reduced to 28 genes with a fold-change (FC) cutoff >10, and these were considered DEGs. Among the DEGs, 13 genes were upregulated and 15 genes were downregulated (Table 2).

**Figure 1:**
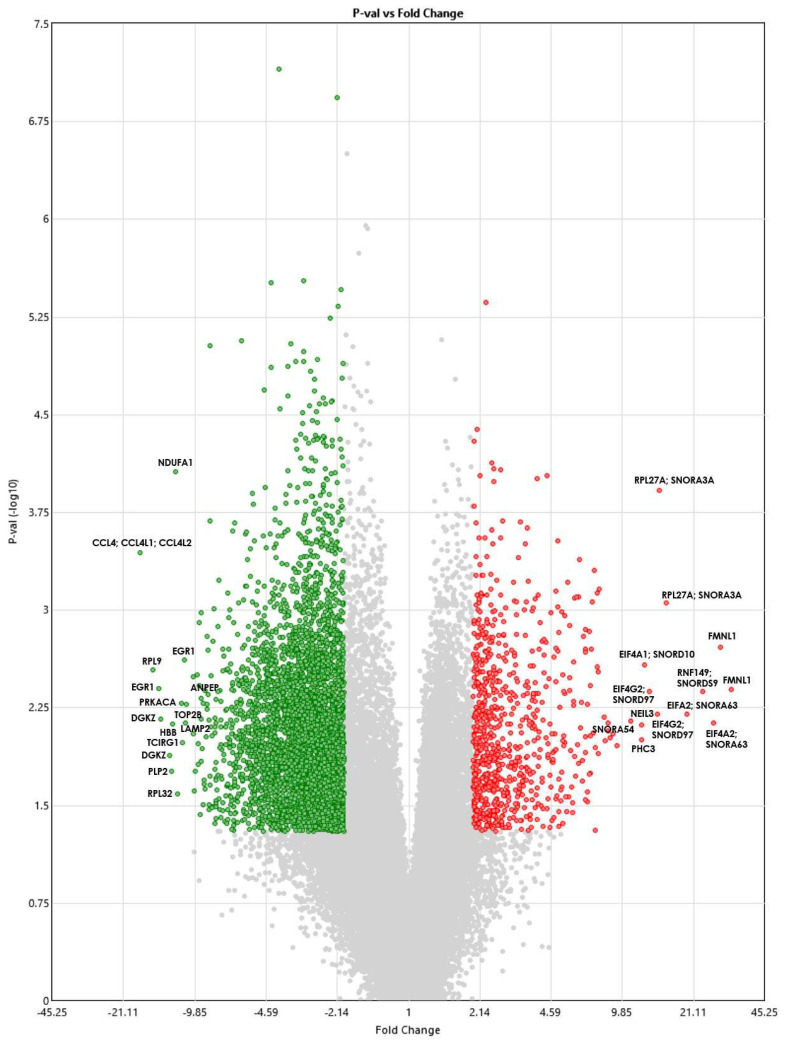
Volcano plot showing DEGs between TAO patients and healthy controls using the Transcriptome Analysis Console version 4.0.1.36 software. The ANOVA *p*-values (shown on the *y*−axis) are log10 transformed, and the fold change (shown on the *x*−axis) is linear. Red indicates upregulated genes, and green indicates downregulated ones.

Figure 1 shows a volcano plot showing the DEGs. The red dots show genes that are significantly upregulated, whereas the green dots show genes that are downregulated. The gray dots represent genes that are not statistically significantly different between the TAO patients and controls.

The distribution of the fold changes of DEGs and transcripts is shown in Table 2. A fold change of ≤2 or >2 was taken for the 4970 DEGs. Then, the 28 genes with fold changes of −10 or >10 (*p* < 0.05) were selected for GO analysis to investigate the potential changes at the molecular level that are both large in scale and stable over time.

### 3.2. Gene Ontology (GO) Enrichment Analysis for the 28 DEGs

An enrichment analysis was performed for gene ontologies, pathways, and domains. This shows the term is richer in the protein set in the network than in the background. We associated the 28 genes as functional categories (GO biological process, GO cellular component, and GO molecular function) through the ShinyGO v0.741 database (http://bioinformatics.sdstate.edu/go74/ accessed on 20 December 2023) via enrichment analysis. The false discovery rate (FDR) was calculated using the nominal *p*-value (≤0.05) obtained from the hypergeometric test. The fold enrichment was determined by dividing the genes of a pathway in the list by the background percentage. There is a tendency for FDRs to be smaller for large pathways due to increased statistical power. This measure is used to indicate how strongly certain genes are overrepresented within a pathway.

**Figure 2:**
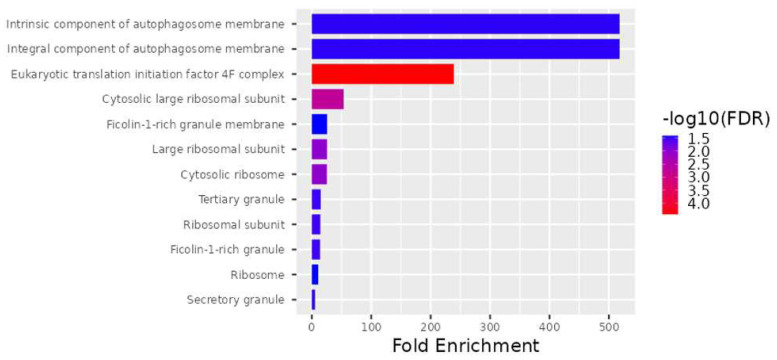
Significantly increased levels for the 28 DEG cellular components.

**Figure 3:**
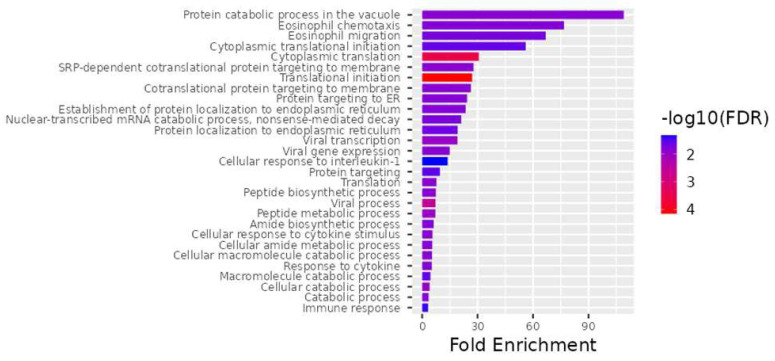
The top 28 differentially expressed genes (DEGs) for the best biological processes. ShinyGO v0.741, a web−based program, was used for all gene ontology enrichment studies.

According to the FDR q-value and *p*-value, the cellular components significantly enriched by the 28 DEGs are shown in Figure 2, and the biological processes are shown in Figure 3. Among the most common cellular components are the eukaryotic translation initiation factor 4F complex (GO:0016281), the cytosolic large ribosomal subunit (GO:0022625), the large ribosomal subunit (GO:0015934), the cytosolic ribosome (GO:0022626), the ribosomal subunit (GO:0044391), the tertiary granule (GO:0070820), the ficolin-1-rich granule (GO:0101002), the secretory granule (GO:0030141), the intrinsic component of autophagosome membrane (GO:0097636), the integral component of autophagosome membrane (GO:0097637), the ribosome (GO:0005840), and the ficolin-1-rich granule membrane (GO:0101003) (Figure 2).

An examination of biological processes uncovered the processes translational initiation (GO:0006413), cytoplasmic initiation (GO:0002181), viral process (GO:0016032), peptide metabolic process (GO:0006518), viral transcription (GO:0019083), cellular catabolic process (GO:0044248), translation (GO:0006412), cotranslational protein targeting to membrane (GO:0006613), SRP-dependent cotranslational protein targeting to membrane (GO:0006614), protein catabolic process in the vacuole (GO:0007039), catabolic process (GO:0009056), viral gene expression (GO:0019080), peptide biosynthetic process (GO:0043043), cellular amide metabolic process (GO:0043603), cellular macromolecule catabolic process (GO:0044265), cellular response to cytokine stimulus (GO:0071345), response to cytokine (GO:0034097), protein targeting to ER (GO:0045047), establishment of protein localization to endoplasmic reticulum (GO:0072599), amide biosynthetic process (GO:0043604), eosinophil chemotaxis (GO:0048245), nuclear-transcribed mRNA catabolic process, nonsense-mediated decay (GO:0000184), eosinophil migration (GO:0072677), protein localization to endoplasmic reticulum (GO:0070972), cytoplasmic translational initiation (GO:0002183), macromolecule catabolic process (GO:0009057), protein targeting (GO:0006605), immune response (GO:0006955), and cellular response to interleukin-1 (GO:0071347) as being important (Figure 3).

**Figure 4:**
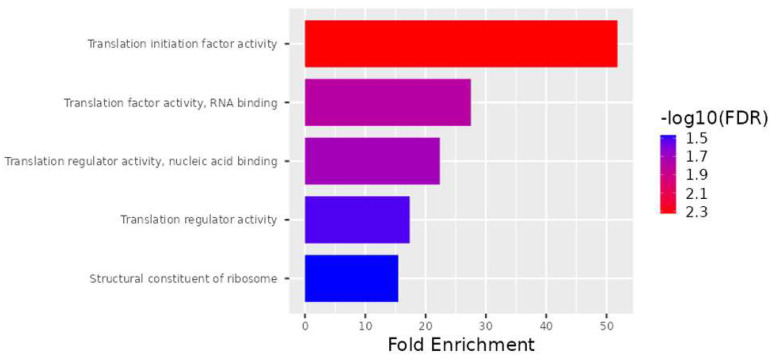
Schematic representation of the molecular functions mediated by gene products. Actions performed by molecules such as proteins and RNA are referred to as molecular functions. The top 28 DEGs are ranked according to molecular function as well as fold enrichment.

As part of the analysis of molecular function, the translation initiation factor activity, translation factor activity, RNA binding, translation regulator activity, nucleic acid binding, translation regulator activity, and structural constituents of ribosomes were determined to be enriched in the subset of genes (Figure 4).

**Figure 5:**
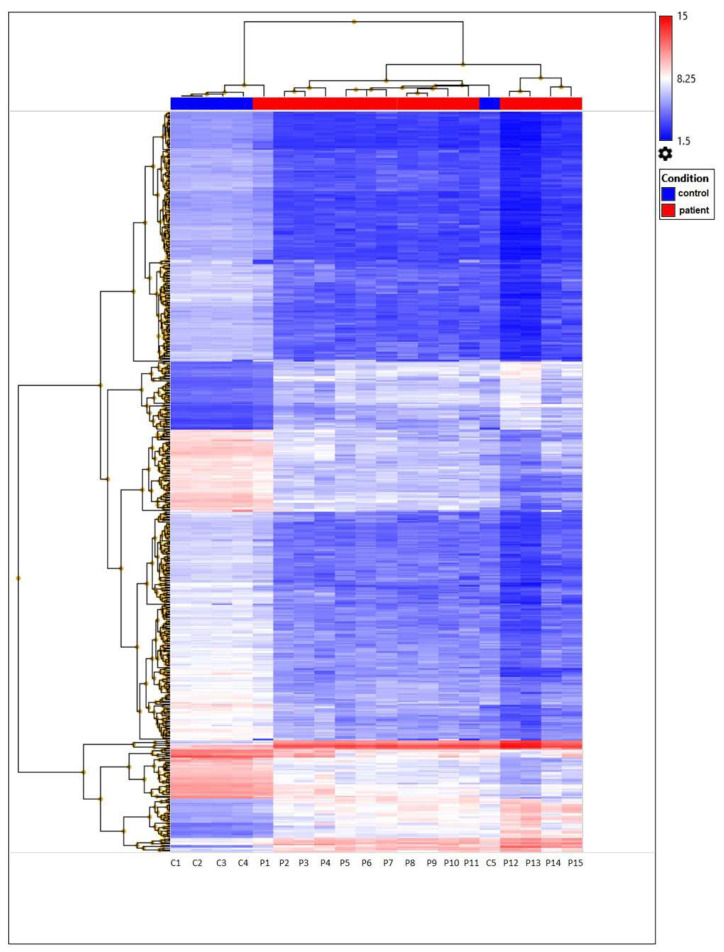
Unsupervised hierarchical cluster analysis based on the Clariom D microarray expression data of 477 DEGs from TAO patient and control samples. Those expressed at high levels are shown in red, whereas those at the lowest levels are indicated in blue.

**Figure 6:**
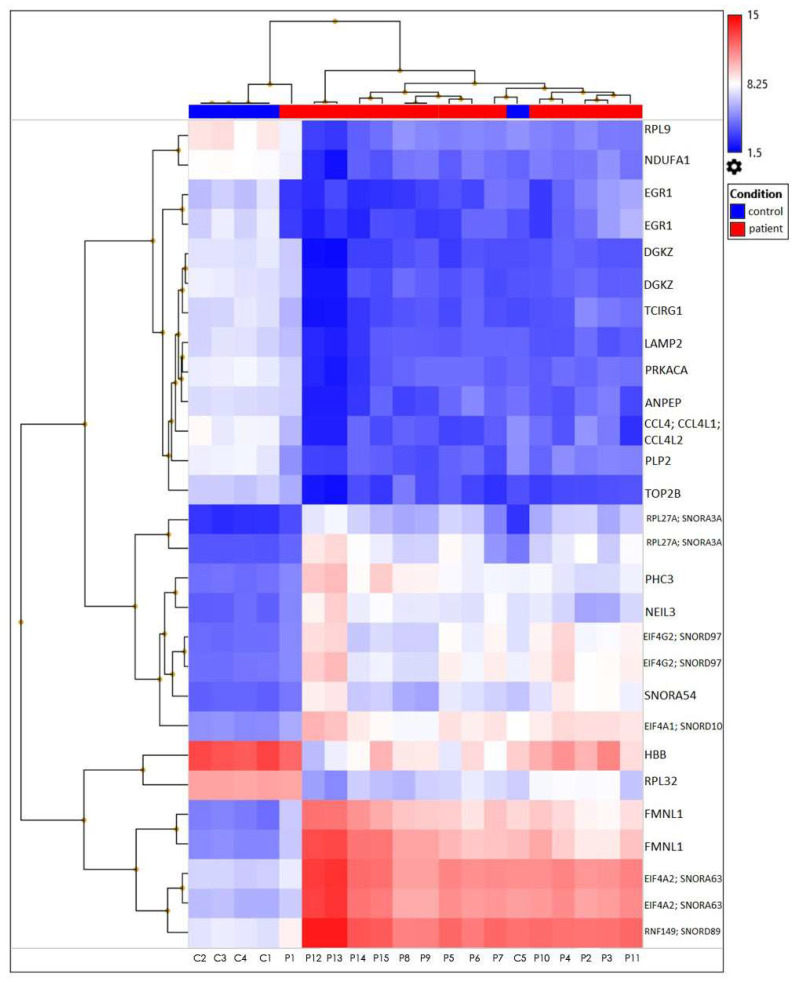
A heatmap plot of the 28 differentially expressed genes from TAO patients versus healthy controls. These two clinical characteristics have distinct expression patterns at the level of gene expression.

The heatmap represents filtered cluster analysis for those genes for which the reliability of differences is highly important (*p* < 0.05). The resulting DEGs are clustered according to the patient and control samples. Each gene is represented by a row, and each sample is represented by a column. The log2 of the relative expression levels is shown as colors (blue represents low relative expression, whereas red represents high relative expression) (Figure 5 and Figure 6).

**Figure 7:**
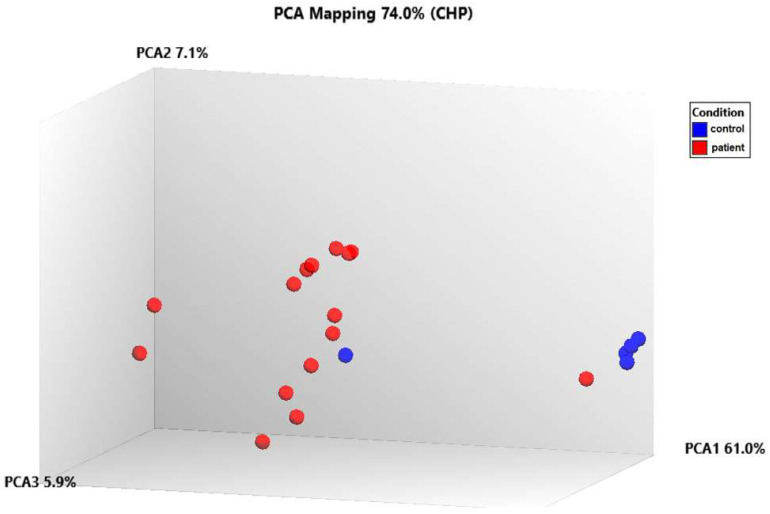
Analyzing the transcriptome data using principal component analysis (PCA).

The data was first normalized, then analyzed using principal component analysis (PCA) to track how the samples were distributed. A comparison of the patient and control samples’ gene expression levels is shown using a three-dimensional scatter plot. The fifteen patient samples were included in one group and the five control samples were put in another based on PCA (Figure 7). We found that the gene expression levels of some genes in control number 5 were different than the other controls. This analysis was performed using PCA, a method that reduces the number of dimensions by keeping the data set with the most variation. We determined that the *RPL27A*, *SNORA3A*, *EIF4G2*, *SNORD97*, *SNORA54,* and *NEIL3* genes were downregulated in a similar way to the other controls but their expression levels differed. However, in control number 5, the expression levels of the *EIF4A2*, *SNORA63*, *RNF149*, *SNORD89,* and *FMNL1* genes were observed to be upregulated, just like in patients with TAO. Since extensive data is obtained as a result of transcriptome analysis, there may be differences in the gene expression levels due to the biological and clinical heterogeneity in the samples. On the other hand, although they were excluded from TAO and other vascular diseases as a result of the tests and examinations performed in the control groups we included in the study, it can be speculated that control number 5 may have a genetic risk for vascular diseases.

### 3.3. Network of DEG Protein–Protein Interactions

**Figure 8:**
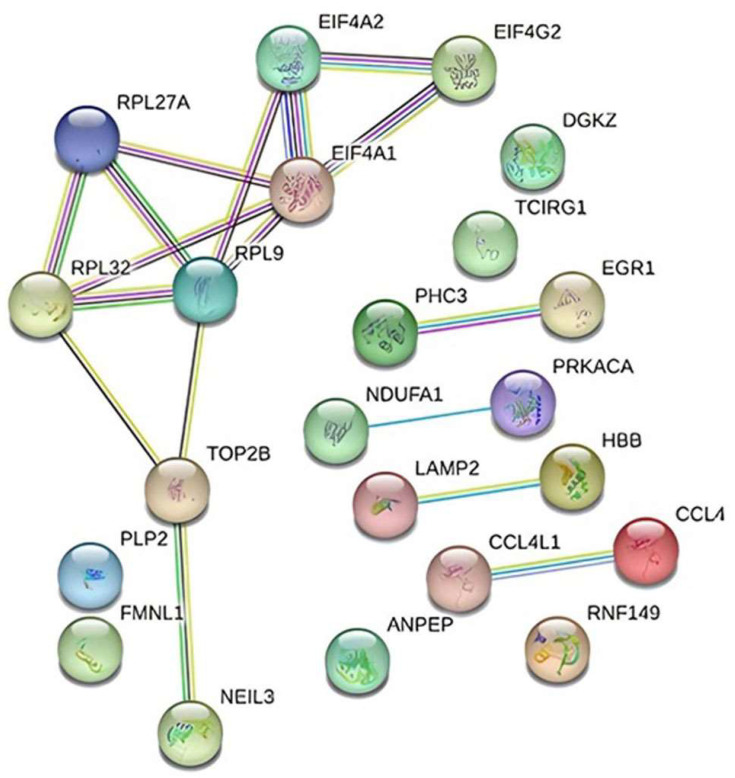
Interaction diagram depicting the 22 genes found to be differentially expressed in TAO patients using the String v11.5 database.

STRING was used in conjunction with Cytoscape to generate the PPI network of gene interactions. The final structure of the network consists of 22 nodes and 17 edges (Figure 8).

Gene–gene and network interactions were constructed using STRING v11.5 analysis (https://string-db.org/, accessed on 20 December 2023). Our preliminary examination of TAO patients utilizing transcriptome profiling arrays identified 22 genes whose expression levels varied. STRING, a web-based tool used for network analysis, was applied to the likely connections of these 22 genes and the resultant network’s interactions were found to be enriched for biologically meaningful relationships (PPI enrichment *p*-value: 0.00491). Using the genomic association method, the Cytoscape plugin selected the five highest-scoring genes (*RPL9*, *RPL27A*, *RPL32*, *EIF4G2*, and *EIF4A1*) from the network (Table 3).

Based on our findings, protein-level investigations and validation of the *RPL9*, *RPL27A*, *RPL32*, *EIF4G2*, and *EIF4A1* genes may be effective in the treatment of TAO disease.

## 4. Discussion

TAO is an inflammatory condition that mostly affects the medium-sized and tiny arteries in the limbs. Despite it having been widely found to be related to cigarette smoking, the precise etiology of TAO is unknown [26,27]. The internal elastic lamina of the afflicted artery is preserved throughout all three stages of TAO, unlike in other types of vasculitis. Endothelial cells initiate and sustain the inflammatory response and endothelium-dependent vasorelaxation is hindered in forearm blood flow investigations. Although the cause and mechanism of TAO are unknown, smoking has been associated with its development and worsening in earlier research [27]. Normal or negative results are often shown for TAO serologic indicators (increased levels of acute phase reactants such as the Westergren sedimentation rate and C reactive protein, increased levels of circulating immune complexes, and normal or decreased levels of autoantibodies such as antinuclear antibody, rheumatoid factor, complement levels, etc.) [28]. Even though cigarette smoking is known to increase the likelihood of developing TAO, hereditary factors may also play a part in the disease’s development [29]. There was no correlation between having a homozygous genotype for the *MTHFR* gene polymorphism and TAO risk; hence, this variant was ruled out as a potential causal factor [30]. Endothelial cells have high levels of toll-like receptor-4, and when these cells become active, they start a response pathway that depends on myeloid differentiation factor 88 (*MyD88*) [31]. The rs7744 reference SNP in the 3′-untranslated region of the *MyD88* gene has been linked to thymic agranulocytosis [32,33]. According to immunogenetic analyses, the HLA-related gene associated with TAO susceptibility is connected to the HLA-B54-MICA-1.4 haplotype [34]. Masoudian et al. investigated the role of polymorphisms related to vascular endothelial dysfunction in the progression of TAO and discovered that eNOS-T786C and PAI-1 (4G/5G) were two of the most significant ones [35].

miR-100 inhibits chronic inflammation in TAO, a segmental inflammatory disease that is not atherosclerotic. Wang et al. discovered that overexpressing miR-100 in H_2_0_2_-induced human umbilical vein endothelial cells suppressed matrix metalloproteinase-9 expression. MiR-100 has also been demonstrated to suppress H_2_O_2_-induced inflammation, oxidative stress, and cell death in human umbilical vein endothelial cells by targeting the Notch signaling pathway [36].

A compatible history, supporting physical findings, and definitive vascular abnormalities on imaging examinations are needed to diagnose TAO. Thromboangiitis obliterans has been diagnosed using a variety of criteria. The diagnostic criteria for TAO have been developed. Age < 45 years, current or recent cigarette use, distal extremity ischemia verified by noninvasive testing, exclusion of thrombophilia, autoimmune illness, diabetes, and proximal emboli, and consistent angiographic findings are the common clinical criteria [37]. TAO may include microvessels surrounding the sympathetic ganglia and their inflammatory cells that extend into the ganglia like neural inflammation in the peripheral neurovascular bundle. This disease may be responsible for the genesis of sympathetic ganglion inflammation in TAO, as well as neurogenic inflammation, which causes a general vasoconstriction and even vascular inflammation and may raise the risk of thrombotic events by activating platelets [38]. Tobacco abstinence appears to be the only treatment for TAO that is generally acknowledged [39]. When it comes to treating damaged arteries, endovascular procedures are increasingly favored over bypass surgeries [40]. TAO patients with significant lower limb ischemia may revascularize with modern endovascular treatments [41]. Ischemic ulcers and chronic pain are two examples of the severe consequences that may be treated with stem cell treatment [42]. To this day, amputation is the only effective method of treating the condition [43,44]. Gene therapy employing angiogenic growth factors such as vascular endothelial growth factor (VEGF) has been developed as a novel cardiovascular disease treatment due to advances in molecular biology [45]. VEGF, which plays a crucial regulatory role in angiogenesis and angiopoiesis, significantly stimulates the proliferation of vascular endothelial cells (VECs) [46].

TAO is connected to the immune system through oxidative stress, damage to T cells, the release of interleukin-33 (IL-33), MyD88-dependent TLR signaling, inflammation in the sympathetic ganglia, the COX inflammatory pathway, IFN-γ, VEGFR1, and HMGB-1. Recognizing the molecular processes that cause TAO may lead to innovative molecular therapeutics that restore the immunologic balance. It has been suggested that oxidative stress plays a role in the progression of TAO. In contrast with healthy smokers and non-smokers, TAO patients have a much higher amount of oxidative stress. Activation of the endothelial nuclear-factor-kappa-B-inducible nitric oxide synthase (NF-κB-iNOS-NO) pathway also makes oxidative stress more likely. This leads to more nitric oxide being made, which, when mixed with the superoxide anion, can strongly oxidize the nitrite anion. Direct endothelial dysfunction and lipid peroxidation result from this. In the process of controlling allergic inflammation, it was shown that endothelial cells are the most important functional target for IL-33. If the IL-33/ST2 pathway is changed, it could become a new therapeutic target for treating or stopping the different inflammatory events that happen in TAO. The pathophysiology of TAO and the process of endothelial cell injury are both linked to the *MyD88* single-nucleotide polymorphism in the TLR signaling pathway [47]. Notch signaling is crucial to cardiovascular development and disease. Inflammatory cells from human atherosclerotic lesions displayed significant levels of Notch ligands and receptors. Notch signaling activates inflammatory cells and is linked to inflammation in atherosclerotic lesions. This means that the inflammatory processes in TAO may be linked to the Notch signaling pathway being activated [48]. Because H2O2 made by SOD can activate the VEFG signaling pathway, low SOD activity might prevent angiogenesis, which is a good way to treat TAO. There is evidence that the receptor VEFG-R1 is more highly expressed in both smokers and nonsmokers, meaning that there may be changes in VEFG signaling in TAO patients [49].

Microarray gene expression profiling is a valuable tool for investigating genome-wide expression patterns that are triggered under the studied biological conditions, laying the groundwork for further research into molecular mechanisms and regulatory pathways. As a result, this study compared TAO patients with control groups in order to perform a Clariom D microarray analysis of the gene expression pattern that might be involved in the pathogenesis of TAO. Based on our findings, it is possible to considerably enhance gene sets that may be related to TAO by examining the gene expression in TAO patients with varying severities of disease (Table 2, Figure 2, Figure 3 and Figure 4). In our study, the downregulated genes associated with TAO were identified as *PLP2*, *CCL4*, *CCL4L1*, *CCL4L2*, *EGR1*, *RPL9*, *LAMP2*, *PRKACA*, *TOP2B*, *DGKZ*, *ANPEP*, *TCIRG1*, *NDUFA1*, *HBB*, and *RPL32*. Additionally, the *RPL27A*, *SNORA3A*, *FMNL1*, *EIF4A1*, *SNORD10*, *RNF149*, *SNORD89*, *EIF4G2*, *SNORD97*, *EIF4A2*, *SNORA63*, *SNORA54*, *PHC3*, and *NEIL3* genes were determined to be upregulated (Table 2).

In a case–control study, Arvind et al. examined the possible genes and pathways related to coronary artery disease (CAD) by using global expression profiling in conjunction with biological network analysis. The results pointed to the possibility that inflammatory genes and cell regulatory genes interact and jointly contribute to the onset of CAD. Strong correlations were found between the genes *CCL4*, *DDX58*, and *CDC42*, as well as between genes known to influence cell mitogenesis and differentiation, such as *EGR1* and *RGS1*, which are involved in the regulation and proliferation of B cells. Arvind et al. found that in cases of CAD, the *CCL4* gene was significantly upregulated compared with controls after adjusting for age, sex, and statin use [50]. In our study, we found that the *CCL4* gene was downregulated in cases of TAO.

The atherosclerosis-related *CCL4* gene is one of the ligands for the C-C chemokine receptor ligand type 5. It is possible that vascular inflammation in atherosclerosis is influenced by T-cell adaptive immunity, as particular immune responses may modify atherosclerosis by targeting plaque antigens such ox-LDL. *CCL4* can produce reactive oxygen species to help THP-1 cells, which are human monocytic cells, stick to human endothelium cells in a lab setting. In clinical practice, atherosclerosis is associated with elevated levels of circulating *CCL4*. *CCL4* is found in atherosclerotic plaques on T cells, smooth muscle cells, and macrophages and is much more highly expressed in susceptible plaques [51].

Some of the functions that *FMNL1* is involved with include migration, membrane protrusion creation, and macrophage phagocytosis. The fact that Jurkat T cells move the centrosome to the immunological synapse and the Golgi apparatus structure has been linked to *FMNL1*. *FMNL1* may also play a part in the cytotoxic activity of CD8 T cells. Research has shown that in autoimmune conditions, T cells may upregulate *FMNL1* expression via transcription. However, in primary lymphocyte motility, trafficking, and transendothelial migration, *FMNL1*’s function and possible method of action remain unclear [52].

*EGR1*, a master transcriptional regulator, controls vascular issues when proangiogenic stimuli and high blood sugar occur in the retinal endothelium. In vascular diseases and diabetes, there is an increase in the EGR1 protein. Vascular problems such as atherosclerosis, inflammation, coagulation, ischemia, and reperfusion damage have been linked to higher levels of *EGR1* and the genes it targets [53]. In many models of vascular disorders, researchers have shown an increase in the levels of a zinc finger transcription factor called *EGR1*. Several hormonal and environmental factors, such as cytokines, growth factors, reactive oxygen species, high blood sugar, and stress caused by stretching, may raise *EGR1* levels in the blood vessels. A significant signaling cascade called the mitogen-activated protein kinase pathway controls *EGR1* transcription. The targeted removal of *EGR1* using DNAzymes, antisense oligonucleotides, or RNA interference has also helped to elucidate what role it plays in the development of vascular disease [54].

Ribosomal protein L9, also known as RPL9, is a component of the 60S subunit of the ribosome, which is the part of the ribosome that is responsible for translating mRNA into proteins. Researchers have shown that *RPL9* gene mutations may affect translation, leading to platelet dysfunction. Ribosomal-related genes such as *RPL31* and *RPL9* may be implicated in the pathogenesis of systemic vasculitis, which is thought to be uncontrolled genetics that promotes Takayasu arteritis through controlling ribosome pathways [55]. Cardiovascular diseases are linked to platelet activation and thrombotic events at afflicted vascular locations. The mean platelet volume (MPV) was found to be significantly higher in TAO patients and has diagnostic importance. Our study suggests that the downregulation of *RPL9* is associated with platelet dysfunction. Silverstein and Febbraio showed that surface expression of LAMP1 requires platelet stimulation by a potent agonist. Platelets activated with high doses of the mild agonists ADP or epinephrine almost completely lacked the thrombin-induced increase in surface expression of LAMP2 [56]. We found that the expression of LAMP2 expressed on the surface of activated platelets is downregulated.

Patients with coronary artery disease showed an upregulation of LAMP2 expression in peripheral blood mononuclear cells when compared with the control group. The collected data revealed a role for LAMP2 in lipid homeostasis and coronary artery disease development [57]. Atherosclerosis progression is accelerated in NEIL3-deficient individuals via activation of the Akt signaling pathway and other non-canonical processes impacting the phenotype of vascular smooth muscle cells [58].

Cheng et al. discovered that SNORA54 is overexpressed in both ischemic stroke (IS)-current smokers and control smokers when compared to IS-never smokers and control never smokers, respectively. It has been suggested that SNORA54, which is expressed in smokers both before and after a stroke, may be related to the chance of having a stroke [59]. Plaque rupture and subsequent deposition occur in cycles, leading to eventual artery closure. Therefore, the atherosclerotic zone may be under the control of new macrophage production and activation through the differential expression of the cell-proliferation-related *RPL27A* gene [60]. It can be said that the upregulation of *RPL27A* in our study may be related to the initiation of the angiogenesis process as a result of increased cytokine levels and the stimulation of macrophages in ischemia tissues. The upregulation of miR-24-3p by silencing the long non-coding RNA MCM3AP antisense RNA 1 reduces EIF4G2 and speeds up the proliferation and migration of vascular endothelial cells in rats with myocardial infarction. The overexpression of *EIF4G2* has been reported to promote inflammation [61]. This explains the upregulation of the *EIF4G2* gene in our study, which explains the inflammation of blood vessels in TAO disease.

Eyster et al. evaluated global gene expression patterns in the common iliac arteries of monkeys with varying degrees of atherosclerosis and found that *RNF149* was increased in atherosclerotic arteries [62]. In our results in TAO (a vascular disease that involves medium and small vessels without atherosclerosis and causes narrowing and occlusions as a result of the inflammation of arteries and veins) patients, we detected an increase in *RNF149* expression. Zhong et al. found that *EIF4A1* expression was upregulated in the aortic wall tissues of patients with acute type A aortic dissection, a catastrophic disease with high mortality but whose pathogenesis is not fully elucidated [63]. Similarly, we found that *EIF4A1* expression was upregulated in TAO disease with fine arterial occlusions.

The mean log^2^FC values of 28 statistically significant (*p* < 0.05) genes from 477 differentially expressed genes were calculated by applying a one-way cross-subject ANOVA test consisting of TAO patients and healthy controls (Figure 6). Using principal component analysis (PCA) to show the distribution of samples, the 15 patient samples were included in one group, whereas the 5 control samples were included in another group (Figure 7). Next, the DEGs interacting through a protein–protein interaction network were identified in STRING (Figure 8, Table 3).

Compared with healthy controls, *RPL27A*, *FMNL1*, *EIF4A1*, *RNF149*, and *EIF4G2* were upregulated in TAO patients, whereas *PLP2*, *CCL4*, *EGR1*, *RPL9*, *LAMP2*, and *DGKZ* were downregulated, suggesting the close association of these genes with TAO progression. We hypothesize that our results will help shed light on the disease’s pathophysiology by illuminating discrepancies between the transcript levels of genes not directly linked to TAO and the transcripts of these genes. We believe that these new candidate biomarkers, which we validated from transcriptome data, will be beneficial in terms of individualized medicine and treatment, especially since there is no complete cure for TAO today and new targeted therapies are needed.

Although it affects both genders and all races, TAO’s incidence is much higher in the male gender. No epidemiological study has been found in the literature on the incidence of TAO in the Turkish population. In addition, our transcriptome study is the only study specific to Turkish society and has the feature of contributing to the literature for the first time.

The limitations of the study include that the 10-fold misregulated genes were not confirmed at the tissue level. Confirming these genes at the tissue level in the continuation of the study will be beneficial in elucidating the pathogenesis of TAO.

## 5. Conclusions

No epidemiological study has been found in the literature on the incidence of TAO in the Turkish population. In addition, our transcriptome study is the only study specific to Turkish society and has the feature of contributing to the literature for the first time. Therefore, as a continuation of this study, changes or modifications in the protein levels of the genes that we found to be significant for TAO disease can be determined by various methods used in proteomics technology.

## Figures and Tables

**Table 1:** Biochemical index and ABI distributions of TAO patients.

	Mean (SD)	Median (Min–Max)
Age (Year)	45.47 (6.94)	44.00 (31–57)
Age at diagnosis (Year)	37.4 (9.80)	36.00 (23–56)
Smoking (pack/year)	23.2 (5.44)	24 (15–33)
Patients who quit smoking (year of quitting) (n = 7)	6.43 (6.45)	4 (1–18)
Patients who continue to smoke (pieces/day) (n = 8)	11.75 (8.05)	12.5 (2–20)
Lower ABI	76.00 (9.86)	80.00 (60–100)
Upper ABI	119.33 (19.44)	120.00 (90–160)
Prothrombin activity	101.01 (18.77)	102.50 (41.30–127.20)
Factor V	116.71 (29.04)	122.50 (71–157)
Blood glucose (mg/dL)	101.80 (33.10)	92.00 (81–208)
LDL (mg/dL)	130.00 (29.74)	130.00 (78–186)
HDL (mg/dL)	38.27 (11.15)	35.2 (15.9–57)
TG (mg/dL)	190.09 (108.52)	160.50 (68.8–432.5)
Proteins S	89.02 (32.47)	80.40 (46.4–148.5)
Protein C	108.61 (26.43)	119.10 (40.9–137.20)
Cardiovascular risk percentage	7.80 (5.36)	6 (3–18)

TAO: thromboangiitis obliterans; ABI: ankle brachial index; LDL: low density lipoprotein; HDL: high density lipoprotein; TG: triglyceride; SD: Standard deviation.

**Table 2:** Top 28 downregulated and upregulated genes.

ID	Patient Avg (log2)	Control Avg (log2)	Fold Change (FC)	*p*-Value	FDR *p*-Value	Gene Symbol	Description
11723032_a_at	4.22	7.82	−12.08	8.70 × 10^5^	0.0409	PLP2	proteolipid protein 2 (colonic epithelium-enriched)
11757158_x_at	6.69	2.81	14.68	0.0001	0.0460	RPL27A; SNORA3A	ribosomal protein L27a; small nucleolar RNA, H/ACA box 3A
11718982_s_at	3.83	7.98	−17.75	0.0004	0.0604	CCL4; CCL4L1; CCL4L2	chemokine (C-C motif) ligand 4; chemokine (C-C motif) ligand 4-like 1; chemokine (C-C motif) ligand 4-like 2
11757157_at	7.79	3.8	15.8	0.0009	0.0714	RPL27A; SNORA3A	ribosomal protein L27a; small nucleolar RNA, H/ACA box 3A
11763689_x_at	9.64	4.82	28.22	0.0019	0.0841	FMNL1	formin like 1
11752940_a_at	3.34	6.8	−10.97	0.0024	0.0864	EGR1	early growth response 1
11748875_a_at	8.92	5.28	12.48	0.0026	0.0881	EIF4A1; SNORD10	eukaryotic translation initiation factor 4A1; small nucleolar RNA, C/D box 10
11715238_x_at	4.93	8.88	−15.45	0.0029	0.0893	RPL9	ribosomal protein L9
11717479_at	3.96	7.28	−10.04	0.0032	0.0905	LAMP2	lysosomal-associated membrane protein 2
11717860_a_at	3.48	7.34	−14.5	0.0040	0.0938	EGR1	early growth response 1
11763688_at	10.11	5.13	31.65	0.0041	0.0942	FMNL1	formin like 1
11757177_s_at	12.12	7.57	23.4	0.0042	0.0946	RNF149; SNORD89	ring finger protein 149; small nucleolar RNA, C/D box 89
11757169_x_at	8.09	4.37	13.18	0.0042	0.0947	EIF4G2; SNORD97	eukaryotic translation initiation factor 4 γ, 2; small nucleolar RNA, C/D box 97
11757881_s_at	4.27	7.78	−11.39	0.0052	0.1002	PRKACA	protein kinase, cAMP-dependent, catalytic, α
11716247_s_at	3.44	6.87	−10.8	0.0053	0.1003	TOP2B	topoisomerase (DNA) II β
11757121_x_at	11.32	7.03	19.58	0.0063	0.1044	EIF4A2; SNORA63	eukaryotic translation initiation factor 4A2; small nucleolar RNA, H/ACA box 63
11757168_at	8.38	4.54	14.3	0.0063	0.1044	EIF4G2; SNORD97	eukaryotic translation initiation factor 4 γ, 2; small nucleolar RNA, C/D box 97
11731346_a_at	3.69	7.52	−14.2	0.0069	0.1066	DGKZ	diacylglycerol kinase, zeta
11757163_at	7.54	4.11	10.74	0.0071	0.1074	SNORA54	small nucleolar RNA, H/ACA box 54
11717127_a_at	3.84	7.28	−10.86	0.0073	0.1080	ANPEP	alanyl (membrane) aminopeptidase
11757120_at	11.06	6.34	26.28	0.0073	0.1080	EIF4A2; SNORA63	eukaryotic translation initiation factor 4A2; small nucleolar RNA, H/ACA box 63
11755105_a_at	3.95	7.59	−12.49	0.0076	0.1091	DGKZ	diacylglycerol kinase, zeta
11722281_a_at	8.11	4.51	12.11	0.0077	0.1094	PHC3	polyhomeotic homolog 3 (Drosophila)
11740012_a_at	7.64	4.03	12.16	0.0099	0.1171	NEIL3	nei-like DNA glycosylase 3
11744180_a_at	3.78	7.28	−11.29	0.0104	0.1179	TCIRG1	T-cell, immune regulator 1, ATPase, H+ transporting, lysosomal V0 subunit A3
11753867_a_at	4.56	8.25	−12.92	0.0130	0.1254	NDUFA1	NADH dehydrogenase (ubiquinone) 1 α subcomplex, 1, 7.5kDa
11715347_s_at	9.28	12.94	−12.63	0.0174	0.1378	HBB	hemoglobin, β
11744336_s_at	7.16	10.72	−11.83	0.0258	0.1573	RPL32	ribosomal protein L32

**Table 3:** Five highly interconnected genes were predicted by Cytoscape based on score values.

node1	node2	node1 Accession	node2 Accession	Score
RPL9	RPL27A	ENSP00000400467	ENSP00000346015	0.999
RPL9	RPL32	ENSP00000400467	ENSP00000416429	0.999
RPL32	RPL27A	ENSP00000416429	ENSP00000346015	0.999
EIF4G2	EIF4A1	ENSP00000433664	ENSP00000293831	0.999

## Data Availability

Data are contained within the article.
